# Editorial: Latent Autoimmune Diabetes in Adults (LADA)

**DOI:** 10.3389/fendo.2022.1002776

**Published:** 2022-08-29

**Authors:** Richard David Leslie

**Affiliations:** Immunobiology Department, Queen Mary University of London, London, United Kingdom

**Keywords:** diabetes, autoimmunity, LADA, risk factors, genetics

Since the discovery of insulin one hundred years ago, diabetes was seen as being characterised by hyperglycaemia but constituting different conditions. Initially that heterogeneity was defined as childhood-onset and adult-onset diabetes, then insulin dependent and non-insulin dependent. Realisation that even these distinctions do not identify all such cases lead to the definition morphing into type 1 diabetes and type 2 diabetes, in which the presence of specific diabetes-associated autoantibodies such as to glutamic acid decarboxylase (GADA) and certain histocompatibility (HLA) alleles defining the former by inclusion and the latter by exclusion (1, 2). Unfortunately, GADA is not specific for type 1, insulin-dependent, diabetes and can be identified in cases who do not require insulin treatment at diagnosis. To complicate matters further, it transpires that the commonest presentation of classic type 1 diabetes is in adult-life and, in them, the majority are not, at least initially, insulin dependent. That has led to much discussion about the nature of that disease in adults, its treatment and what it should be called, if not type 1 diabetes. In this sequence of articles on this important topic, leading authorities in the field discuss some of these key questions.

Studies identified heterogeneity within type 1 diabetes including so-called endotypes characterised by the early onset of insulin autoimmunity in cases with HLA DR4 progressing to insulin-dependent diabetes under the age of 7 years (3). Another endotype, characterised by the early onset of GADA in cases with HLA DR3, progress to insulin-dependent diabetes over the age of 13 years and the latter look every similar immunogenetically to adult-onset type 1 diabetes (3, 4). It follows that GADA-positive adult-onset autoimmune diabetes is the commonest form of autoimmune diabetes. The frequency of adult-onset type 1 diabetes in higher in Europe than in South-East Asia, though GADA-positive initially non-insulin requiring diabetes is very similar in China to that to that in Europe (1, 5). Identification of these autoimmune diabetes-associated endotypes has brough some clarity to the confusing nosology which sought to identify adult-onset autoimmune non-insulin dependent diabetes as distinct from classic autoimmune insulin dependent type 1 diabetes. Latent autoimmune diabetes of adults (LADA), slowly progressive insulin dependent diabetes (SPIDDM), slow-onset autoimmune diabetes (SAID), Type 1.5 diabetes and double diabetes are all terms aiming to capture the same disease entity whilst distinguishing the disease from classic type 1 diabetes (1). Whether you see autoimmune diabetes that presents as insulin-dependent, and therefore classic type 1 diabetes, as distinct from a more slowly-progressive form presenting as non-insulin dependent diabetes such as LADA, is academic. Either way the question is why does it differ and what should we do about it?

Hernandez et al. studied a relatively small group of LADA cases and found that major HLA genes associated with type 1 diabetes, that is DRB1*03 and disease-protective HLA alleles, were of similar frequency in LADA and classic adult-onset type 1 diabetes as distinct from type 2 diabetes (6). As predicted from the endotype data, DRB1*04 was less frequent in LADA than type diabetes in line with LADA being a form of late-onset type 1 diabetes (3). Given the widespread use of GADA as a biomarker of LADA in adult-onset diabetes, it is important to recognise that the assay for GADA is not 100% specific and therefore that specificity needs to be increased or other biomarkers are required to make the diagnosis (4, 7). One such is the presence of autoantibodies to insulinoma antigen-2 (IA-2A) which are directed against a fragment of the molecule (IA-2 (256–760) containing both intracellular and extracellular epitopes (4). Tiberti et al. show that it the intracellular domains, which define a cohort with IA-2A and the phenotype of typical LADA and classic type 1 diabetes, namely reduced features of metabolic syndrome (obesity, high triglycerides, low HDL, hypertension) with increased other autoimmune disorders (8, 9). Pan et al. describe the association of LADA with Metabolic Syndrome in detail. While Tian et al. analysed the fatty acid profiles in detail of a mixed cohort of LADA cases and other major formed of diabetes. They found that poly-unsaturated fatty acids were associated with insulin sensitivity and beta cell function, but the associations were insufficient to differentiate the different current categories of diabetes i.e. LADA, type 1 and type 2 diabetes.

Hu et al. focus on recent studies of the intestinal microbiota and how changes could impact the development of LADA. Given the slow disease progression in LADA and the resultant window in which therapies might modify that progression. A detailed analysis by Carlsson describes non-genetic factors which could lead to LADA. Drawing on studies from Scandinavia, she identifies factors linked to type 2 diabetes as predisposing to LADA, namely obesity and smoking. Obvious explanations for such an association include: first, some cases may have false positive GADA, actually having Type 2 diabetes; or second, that autoimmunity and metabolic disturbances can be additive, predisposing to LADA, simply because there is a threshold effect leading to hyperglycaemia and that threshold is lowered by one or the other, or both (7, 9). Intriguingly, she also notes the effect of smoking, which is known to impact another autoimmune disease, rheumatoid arthritis, though the effect is compounded by obesity and the tendency to smoke both being linked to type 2 diabetes. Nevertheless, the interaction with HLA risk alleles and these non-genetically determined risk factors raise the very real possibility that the observation is genuine (6).

Zhao and Li compare and contrast the nature of LADA and rheumatoid arthritis including their respective clinical course and the immune therapy used in the latter (10). Bjorklund et al., having failed to show a beneficial effect in progression of LADA cases with high GADA levels using sitagliptin, tested the feasibility of using intralymphatic GAD-alum given with oral vitamin D as therapy (10); after 5 months, using a limited number of cases, the results were interesting and the study continues. We hope these interesting papers will stimulate the reader to consider LADA in more detail, as we continue to grapple with the management of this important form of diabetes.

## Author contributions

The author confirms being the sole contributor of this work and has approved it for publication.

## Acknowledgments

I appreciate the discussions with my collaborators including the Action LADA consortium, Professor Zhuguang Zhou and Professor Bernhard Boehm.

## Conflict of interest

The author declares that the research was conducted in the absence of any commercial or financial relationships that could be construed as a potential conflict of interest.

## Publisher’s note

All claims expressed in this article are solely those of the authors and do not necessarily represent those of their affiliated organizations, or those of the publisher, the editors and the reviewers. Any product that may be evaluated in this article, or claim that may be made by its manufacturer, is not guaranteed or endorsed by the publisher.
